# A Phase I study of an HLA-DPB1*0401-restricted T cell receptor targeting MAGE-A3 for patients with metastatic cancers

**DOI:** 10.1186/2051-1426-3-S2-P158

**Published:** 2015-11-04

**Authors:** Yong-Chen Lu, Linda Parker, Tangying Lu, Zhili Zheng, Xin Yao, Paul F Robbins, Pierre van der Bruggen, Christopher A Klebanoff, Christian S Hinrichs, Stephanie Goff, Richard Sherry, Udai Kammula, James C Yang, Steven A Rosenberg

**Affiliations:** 1 Surgery Branch / National Cancer Institute / National Institutes of Health, Bethesda, MD, USA; 2 National Institutes of Health, Bethesda, MD, USA; 3 Ludwig Institute for Cancer Research, de Duve Institute, Université Catholique de Louvain. Avenue Hippocrate 74, bte B1.74.03, second floor, Brussels, Belgium; 4 Center for Cancer Research, NCI/NIH, Bethesda, MD, USA; 5 NIH/NCI, Bethesda, MD, USA

## Background

Adoptive transfer of genetically-modified T cells is being explored as a salvage treatment for patients with selected metastatic cancers. Most of the current strategies utilize MHC class I-restricted T cell receptor (TCR) or chimeric antigen receptor (CAR) technologies to genetically modify CD8+ T cells or bulk T cells for patient treatment. Evidence indicates that CD4+ T cells can induce tumor regression, similar to CD8+ T cells. To test this hypothesis, an HLA-DPB1*0401-restricted TCR recognizing MAGE-A3 was isolated from a patient's peripheral blood after MAGE-A3 peptide vaccination. Because HLA-DPB1*0401 is present in 40~70% of the Caucasian population and MAGE-A3 is expressed in up to one third of tumor specimens from a variety of cancer types, this TCR immunotherapy can potentially be beneficial for a significant portion of cancer patients.

## Trial design

Eligible patients were HLA-DPB1*0401 positive with MAGE-A3 positive tumor specimens, and had not responded or had recurred following at least one standard first line therapy for their disease. Patients received a lymphodepleting preparative regimen, followed by adoptive transfer of purified CD4+ T cells transduced with the HLA-DPB1*0401-restricted MAGE-A3 TCR plus systemic high-dose interleukin-2 (IL-2). A cell dose-escalation was conducted, treating 1 patient at each cohort (0.01, 0.03, 0.1, up to 30 billion cells), followed by 6 patients at the highest dose level (100 billion cells). Clinical trial information: NCT02111850.

## Results

To date, 10 patients have been enrolled in this protocol. The latest patient was treated at the highest dose level (100 billion cells). A patient with cervical cancer metastases in her supraclavicular lymph nodes is a confirmed partial responder (PR) by RECIST criteria. Her tumors shrank 85% by 8 months after adoptive transfer of 3 billion TCR-transduced CD4+ T cells. This result demonstrates the safety of administering autologous CD4+ T cells genetically-engineered to express an MHC class II-restricted anti-tumor TCR targeting MAGE-A3 and presents preliminary evidence for efficacy. This clinical trial extends the reach of TCR gene therapy for patients with metastatic cancers. To our knowledge, this is the first genetically-modified CD4+ T cell immunotherapy against cancer.

## Trial registration

ClinicalTrials.gov identifier NCT02111850.

